# 

**Published:** 1893-04-15

**Authors:** 


### CONTENTS.

**School Hygiene** . . . . . . . . . . . . 33

**Around the Hospitals** . . . . . . . . . . 34

**Medical History from the Earliest Times.** By E. T. Withington, M.A., M.B Oxon. I.—The Great Anatomists . 35

**Contemporary Theory and Research** . . . . . . 36

**Nebuchadnezzar's Feast** . . . . . . . . . 37

**Gleanings in Science and Medicine** . . . . . . 38

**Annotations.**—Doctors and Death—A Lesson for Mr. Stead—Stands Edinburgh for Untrained Nurses? . . . . . 39

**Notes and News** . . . . . . . . . . . 40

**Scraps and Gleanings** . . . . . . . . . . 48

**The Book World of Medicine and Science** . . . . viii

**The Student of Medicine** —Appointments, Vacancies . x

**The Hospital Clinic—**

Royal Orthopædic Hospital.—The Treatment of Club Foot . 41

Royal Infirmary, Edinburgh.—The Treatment of Cold Abscess 42

The Practitioners' Bookshelf.—The Factory System of the United States . . . . . . . . . . . . 43

New Drugs, Appliances, and Things Medical —Oswego Prepared Corn—The Magic Cycle and General Oiler—Milk Food for Infants, &c. . . . . . . . . . 44

**The Institutional Workshop—**

Hospital Administration.—Hospital Accounts . . . . 45

Special Journals for Hospital Saturday Funds . . . 46

Practical Departments —Fire Escapes . . . . . . 46

Hospitals of the World. IV.—Nursing Systems Abroad . 47

### EXTRA SUPPLEMENT.—THE NURSING MIRROR.

**News from the Nursing World.**—The Privy Council and the Charter—Charity and Cheerfulness—The Royal National Pension Fund for Nurses—Rest and Recreation—Nursing at Plaistow—Hidden Dangers—Cambridge—Ladies to the Rescue — Practical Sanitation — Successful Canvassing—Reduced Accommodation—A Nurses' Home For Orange, U.S.A.—Short Items—The Disabled Nurse—"The Hospital" Endowed Bed . . . . . . . . . . . . xxi

**Sanitation for Nurses.** By a Sanitary Inspector. I.—Infectious Diseases . . . . . . . . . . xxiii

**Test Questions.**—Given to Nurses of the State Hospital, Utica, New York, prior to their Examination . . . . xxiv

**Minor Appointment** . . . . . . . . . . xxiv

**Australia.**—Prince Alfred Hospital, Sydney . . . . . xxiv

**Everybody's Opinion.**—Women Inspectors—"Attainable Perfection" . . . . . . . . . . . . . xxv

**Notes and Queries—Wants and Workers** . . . . xxv

**For Reading to the Sick.**—Spring Time . . . . . xxv

**Presentation—Hints for Nurses** . . . . . . . xxv

**Our Examination Questions** . . . . . . . . xxvi

**Nursing in Australia** . . . . . . . . . . xxvii

**News, Nurses, and Clubs** . . . . . . . . . xxvii

**Rescued—Appointments** . . . . . . . . . xxviii

**The Book World for Women and Nurses** . . . . xxix

